# Book Reviews

**Published:** 1903-03

**Authors:** 


### BOOK REVIEWS.

A TEXTBOOK OF THE SCIENCE AND ART OF OBSTETRICS. By HENRY J. GARRIGUES, A. M., M. D., Consulting Obsteric Surgeon to the New York Maternity Hospital; Gynecologist to St. Mark's Hospital. Octavo, pp. 874. 504 illustrations. Philadelphia and London: J. B. Lippincott Co. 1902.

This work is divided into two grand divisions,—normal and abnormal. In the normal division, the first part, consisting of six chapters, is termed the foundation. In the second part—twenty-eight chapters—normal pregnancy is described. In the third part—ten chapters—normal labor is dealt with and, in the fourth part—six chapters again—normal puerpery is considered.

Coming now to the second grand division, we find the abnormalities treated,—in the first part abnormal pregnancy in eleven chapters; in the second part abnormal labor in eighteen chapters; in the third part obstetric operations in seventeen chapters; in the fourth part abnormal puerpery in nine chapters; in the fifth part notes on diseases of newborn children in fifteen chapters. This gives the general scheme of the treatise, which is novel, practical, and scientific.

The author takes up every condition, from puberty in the first chapter, to sudden death of the newborn child in the last, and discusses them in detail, but with a conciseness that yet sacrifices nothing to scientific accuracy. Garrigues is first of all scientific, then reduces the science to the practical, and finally divests it of ultra scientific dryness before it goes to the press. In the plan of his book it will be observed that he begins with the simple, passes on to the complicated, and ends with the difficult,—a most admirable basis that appeals alike to student, junior practitioner, and advanced obstetrician.

The author very justly declares that during the past 25 years but four really great improvements in the practice of obstetrics have been made, and these he asserts are antisepsis with its resultant principle—asepsis, the axis-traction forceps, the improved Cesarean section, and the revival of symphyseotomy. While this is quite true, there is yet another important element to consider—namely, the improved methods in teaching obstetrics. Clinical teaching in the lying-in-room has done more to advance the art and science of obstetrics than all else combined, and this author is, happily, one of the ablest exponents of this advance.

Garrigues is a veteran teacher both of gynecology and obstetrics; he has written one of the best treatises on the former, and in the present book, which may be regarded as the crowning work of his life, he has far outstripped his previous record as teacher and author. This work is destined to take place in the front rank as a textbook, and is sure to be referred to in all future time as a scientific expôsition of the obstetric art reduced to the practical and presented in most attractive foim.

The illustrations deserve praise for their number, quality, and artistic perfection. They are profuse, original, some are drawn from nature, several are actual in size, and all are helpful toward a completer understanding of the text.

The distinguished author deserves the congratulations of his professional confrères for the service he has performed toward perfecting the science of medicine.

---

Clinical Surgery. For the Instruction of Practitioners and Students of Surgery. By A. J. Ochsner, B. S., F. R. M. S., M. D., Chicago, Surgeon-in-Chief, Augustana Hospital, and St. Mary's Hospital; Professor of Clinical Surgery, Medical Department, University of Illinois. Royal octavo, pp. 481. Profusely illustrated. Cleveland Press (The Clinical Review Publishing Company). 1902. [Price, cloth, $6.00; half-morocco, $7.00.]

The author of this work has attracted attention of late as a teacher of original methods and of the forceful application of principles. He has constructed a book on these lines, though he modestly declaims originality in his preface. He says he has not invented a single new operation, nor devised a new instrument, but has been content to apply what experience has shown to be the best in surgical practice, past and present. This, after all is about the wisest plan, for it has been said often and truly, that "there is nothing new under the sun." In surgery the most that is new is something described under a new name, or in a new place.

This is essentially a clinical treatise setting forth the author's own methods of procedure in the various conditions dealt with. Under the head of general surgical considerations the author presents a homily on the patient, showing how surgical results are determined often by the individual and his environment. Ochsner then proceeds to discuss in the order as here given, hernia, abdominal surgery, surgery of the gall-bladder, of the stomach, of the intestines, of the kidney, of the pelvis, of the chest, of the neck, of the mouth, of the face, of the skull, of the genitourinary organs, of the rectum, of the female genital tract, and of the extremities. These are the limitations of the book. We will now invite attention to some of its salients.

Some kind friend has sent us a summary of the leading features of this treatise, which is so truthful and concise that we use it in part as a conclusion to the notice.

It calls attention to the following general points: first, the almost life-size of the page illustrations; second, that the engravings are all new, made from original drawings of clinical cases; third, clearness of text and wide margins permitting notes; fourth, excellent quality of paper, press work and binding; fifth, originality, brevity and conciseness; sixth, clinical surgery taught by cases illustrated with cuts and descriptive text; seventh, absence of discussion concerning unsettled problems, elimination of obsolete methods, and avoidance of repetition; eighth, a presentation of newer surgery, and advanced technic, with force and conviction based on experience.

It is a book of instruction for him who must needs practice surgery, and will furnish him an aid and a guide in difficulties that are sure to beset his pathway.

---

The Practice of Surgery. A Treatise on Surgery for the Use of Practitioners and Students. By Henry R. Wharton, M. D., Clinical Professor of Surgery, Woman's Medical College of Pennsylvania; Surgeon to the Presbyterian and Children's Hospitals, Philadelphia. And B. Farquhar Curtis, M. D., Professor of Clinical Surgery, and Adjunct Professor of the Principles of Surgery in the University and Bellevue College of New York. Third edition. Octavo, pp. 1249. Illustrated. Philadelphia and London: J. B. Lippincott Company. 1902.

From the year 1897, when this work first came forth, to the present time, marks an important period in the history of surgery. Into this narrow space, if a section of time may be so designated, events have crowded with an astonishing compactness. Many surgical treatises have issued from the press, each with distinctive features and special merit. From the practical side of the subject, however, it may be asserted that none have excelled the work under consideration.

The aim of the authors at the outset, was to present a book that would contain a reasonably full description of surgical injuries and diseases, to give directions for their treatment, and to outline the accepted facts and theories of etiology, pathology, and bacteriology. How well they have succeeded these three splendid editions in a little more than five years abundantly testify.

Another special aim of the authors has been to present the diagnosis of surgical injuries and diseases in a strong light, and this is a supremely important point, contributing to the great practical character of the treatise; and this, in turn, has made for its success. In this edition, which has not only been revised, but rewritten and reset, will be found a concise yet ample presentation of the practical side of surgery, applicable to the needs of the busy general practitioner. The display subheads make it easy of reference and the mechanical execution of the volume could scarcely be better. In the matter of illustrations it merits great praise for quantity and quality—a matter of great moment to both student and graduate.

The distinguished authors are entitled to a full measure of praise for presenting the profession with one of the most valuable books that has appeared in years, it being a distinct addition to the literature of American surgery.

---

THE PRACTICAL MEDICINE SERIES OF YEAR BOOKS. Ten volumes. Issued under the general editorial charge of GUSTAVUS P. HEAD, M. D., Professor of Laryngology and Rhinology in the Chicago Post-Graduate Medical School. Volume I. General Medicine. Edited by Frank Billings, M. S., M. D., Head of the Medical Department and Dean of the Faculty of Rush Medical College, Chicago, and J. H. Salisbury, M. D., Professor of Medicine, Chicago Clinical School. Duodecimo, pp. 358. Chicago: The Year Book Publishers. 1902. [Price, $1.50; entire series, $7.50.]

This number deals with diseases of the respiratory organs, diseases of the circulatory organs, diseases of the blood and blood-making organs; with general infectious diseases of the ductless glands, diseases of the kidneys, and with a miscellaneous group embracing osteomalacia, osteitis deformans, the caisson disease, and morphine poisoning.

In this book will be found an excellent résumé of the literature of the year relating to the foregoing subjects, supplemented by comments of the editors whenever these seemed necessary to further intelligently exploit the subject commented upon. Whoever desires a practical assembling of the best literature of general medicine for the period indicated will find it in this book.

---

AMERICAN EDITION OF NOTHNAGEL'S PRACTICE. Volume IV. Diseases of the Bronchi and Lungs. Diseases of the Bronchi, by Dr. F. A. Hoffman, of Leipsic. Diseases of the Pleura, by Dr. O. Roseebach, of Berlin. Pneumonia, by Dr. F. Aufrecht, of Magdeburg. Edited, with additions, by JOHN H. MUSSER, M. D., Professor of Clinical Medicine, University of Pennsylvania. Octavo volume of 1,030 pages. Illustrated, including 7 full-page colored lithographic plates. Philadelphia and London: W. B. Saunders & Co. 1902. [Cloth, $5.00 net; half-morocco, $6.00 net.]

The group of monographs that form this volume are upon subjects second in importance to none in the whole domain of medicine. They deal with that portion of the air tract that is extremely vulnerable, and when it becomes diseased it puts the entire economy *hors de combat* for a considerable period of time. These essays, among the ablest ever written upon these subjects, are offered by men of great learning, and who have bestowed exhaustive research upon these several themes.

Without attempting to cover the whole field in a book notice, we may nevertheless, point out some of the novel features developed in the pages of this volume. Passing over the anatomical and physiological additions relating to the bronchi, we note the recording of recent researches on fibrinous bronchitis, on eosinophilia and Fränkel's researches in asthma. The view is upheld that pneumonia is a general pneumococcus infection, in which, of course, toxemia is the chief danger. The surgical treatment of surgical complications is advocated, and modern methods of diagnosis, including Röntgen rays are pointed out, and their useful limitations defined.

The section on pleurisy is particularly attractive, in which is given the newer bacteriological studies relating to this affection; also Morse's recent studies on the leucocytes, together with the other clinical phenomena of greater or less importance.

The additions by the American editor, Dr. Musser, increase the interest and importance of the work in no small degree. By and large, it is a treatise that reflects the highest credit upon the science and literature of medicine.

---

The American Textbook of Obstetrics. In two volumes. Edited by Richard C. Norris, M. D.; Art Editor, Robert L. Dickinson, M. D. Second edition, thoroughly revised and enlarged. Two handsome imperial 8vo volumes of about 600 pages each; nearly 600 text illustrations, and 49 colored and half-tone plates. Philadelphia and London: W. B. Saunders & Co. 1902. [Per volume: cloth, $3.50 net; sheep or half-morocco, $4.00 net.]

The second edition of this work finds the obstetric art advanced considerably beyond its status seven or eight years ago, when the textbook first appeared. It is true, also, that in its scientific aspects obstetrics has progressed amazingly during the last decade. Bacteriology has contributed its full share to this advancement, and chemicobiological research has also added to the improved pathology of the present day. Taking advantage of their opportunities the editors and contributors to this edition have brought it forward in all these as well as in other particulars, until it may be said to express the most advanced thought upon the art and science of obstetrics.

The surgery of obstetrics has been given a deservedly prominent place, and new applications of it have been made; new chapters have been added or old ones rewritten; new illustrations have appeared, and taken together this new edition—which appears in two volumes instead of one, as formerly—presents the whole obstetric field in the strongest light, and boldest manner. Student and practitioner, junior and senior physician—all may gain information of the most valuable kind by studying this work with care. In the art department it is unrivaled and we particularly note that Walcher's position is clearly and properly illustrated.

---

Blakiston's Quiz Compends, No. 4. A compend of Human Physiology. Especially adapted for the use of Medical Students. By Albert P. Brubaker, A. M., M. D., Adjunct Professor of Physiology and Hygiene in the Jefferson Medical College. Eleventh edition, revised and enlarged Illustrated. Philadelphia: P. Blakiston's Son & Co 1902. [Price, 80 cents.]

This compend is one of the most useful of the series, and is an excellent reminder of the essentials of physiology. While it is especially intended for students, it is of almost equal service to practitioners. It will do all of us good to refresh our minds upon this great subject, and this book would be an interesting companion on a railway journey for the purpose named. The table of physiologic constituents at the end of the volume is worth its price.

---

Textbook of Medical Jurisprudence and Toxicology. By John J. Reese, M. D., late Professor of Medical Jurisprudence and Toxicology in the University of Pennsylvania. Sixth edition. Revised by Henry Leffmann, A. M., M. D., Professor of Chemistry and Toxicology in the Woman's Medical College of Pennsylvania. 12mo pp. 676. Philadelphia: P. Blakiston's Son & Co 1902. [Price, $3 00 net.]

The occasional reappearance of this book in new editions is looked forward to by all who are familiar with it, and especially by the students of legal medicine. There have been some recent developments in toxicology, also some of the synthetic organic bodies that are used frequently in the household have been found to occasion poisoning in some instances and these are set forth in this edition. The extensive use of water gas—carbon monoxid—has caused poisoning in numerous instances, it being much more deadly than illuminating gas made in the old way, and this fact is dealt with in this book. Several new phases are noted, among which may be mentioned the employment of alcohol in carbolic acid poisoning. In short, the recent data of trustworthy nature are herein incorporated and the book is thus brought forward to the immediate present in all essential particulars, while still retaining its original distinctive features.

---

The Public and the Doctor. By B. E. Hadra, M. D., Dallas, Texas. 1902. [Price, 50 cents.]

That the relations of the general public and the doctor are not what they should be admits of no argument, because it is a statement that cannot be controverted. This monograph of Dr. Hadra's will serve to bring the people and physicians into closer touch if its circulation and attentive reading should be sufficiently widespread. The eminent position of the author should serve to carry weight to his utterances and to give them a large audience.

